# Availability of Low-Fat Milk and Produce in Small and Mid-Sized Grocery Stores After 2014 WIC Final Rule Changes, Tennessee

**DOI:** 10.5888/pcd14.170008

**Published:** 2017-08-24

**Authors:** David Schlundt, Chiquita Briley, Barbara Canada, Jessica L. Jones, Baqar A. Husaini, Janice S. Emerson, Pamela C. Hull

**Affiliations:** 1 Department of Psychology, Vanderbilt University, Nashville, Tennessee; 2 Department of Family and Consumer Sciences, Tennessee State University, Nashville, Tennessee; 3 Center for Prevention Research, Tennessee State University, Nashville, Tennessee; 4 Department of Medicine, Vanderbilt University Medical Center, Nashville, Tennessee

## Abstract

**Introduction:**

The 2007 Interim Rule mandated changes to food packages in the Special Supplemental Nutrition Program for Women, Infants, and Children (WIC) for implementation by 2009. The 2014 Final Rule required additional changes, including increasing the cash value voucher for fruits and vegetables from $6 to $8 for children by June 2014, and allowing only low-fat (1%) or nonfat milk for mothers and children aged 2 to 4 years by October 2014. This study evaluated the effect of the 2014 Final Rule changes on the food environment of small and mid-sized WIC-authorized grocery stores.

**Methods:**

We analyzed secondary data using a natural experimental design to compare the percentage of shelf space for low-fat and nonfat milk and the number of fresh fruit and vegetable varieties in stock before and after the changes. We collected observational data on 18 small and mid-sized WIC-authorized grocery stores in Nashville, Tennessee, using the Nutrition Environment Measures in Store tool in March 2014 and February 2016.

**Results:**

The mean percentage of shelf space occupied by low-fat and nonfat milk increased from 2.5% to 14.4% (*P* = .003), primarily because of an increase in the proportion of low-fat milk (*P* = .001). The mean number of fresh fruit and vegetable varieties increased from 24.3 to 27.7 (*P* = .01), with a significant increase for vegetables (*P* = .008) but not fruit.

**Conclusion:**

Availability of low-fat milk and variety of fresh vegetables increased after the Final Rule changes in the observed stores. Future research should examine outcomes in other cities.

## Introduction

The Special Supplemental Nutrition Program for Women, Infants, and Children (WIC) was permanently established in 1975 to provide nutrition support to pregnant and lactating low-income women, infants, and children younger than 5 years who are at nutritional risk (eg, poor diet, underweight, anemic). The WIC program offers vouchers for nutritious foods that can be redeemed at WIC-authorized grocery stores and WIC food distribution centers. WIC also provides nutrition education services, breastfeeding support, and referrals for health care and social services (1). In fiscal year 2015, more than 8 million families participated in WIC and received more than $4 billion dollars in direct food assistance; the average family benefit was $43.37 per month (2).

The goal of the WIC program is to improve health outcomes in women and their infants who are at risk because of inadequate nutrition (3). In recent years, the value and kinds of food provided via WIC vouchers have been adjusted to promote breast feeding and healthier food choices (4,5). The WIC Interim Rule, approved in 2007, mandated changes to WIC food packages, which states were required to implement by October 2009. These changes included reducing amounts of milk, cheese, eggs, and juice; adding whole grains; adding a cash value voucher (CVV) for fruits and vegetables, with the goal of increasing both the quantity and variety of fruit and vegetable consumption; and allowing some options for substituting foods (6). The WIC Final Rule, approved in March 2014, required the following additional changes: increasing the CVV for children from $6 to $8 by June 2, 2014; allowing only low-fat (1%) or nonfat milk for mothers and children aged 2 to 4 years; and allowing greater flexibility for authorizing soy-based beverages and tofu as milk substitutions for children (7). Several studies have examined the effect of the 2009 package changes on food availability in WIC-authorized grocers and on dietary intake among WIC participants (8–14). However, no research has examined the effect of the 2014 changes.

Increasing the availability of healthy foods can lead to increased purchases of healthy foods among low-income populations (15,16). Low-income women often live in neighborhoods with poor access to full-service grocery stores (17). Healthy food options are often more difficult to find in small neighborhood stores than in large full-service grocery stores (18). Small stores may find it challenging to offer the same variety of foods as larger grocery stores (19). In evaluating changes to the WIC program, it is important to examine small and mid-sized stores in low-income areas (20). The objective of our study was to evaluate changes in the availability of low-fat milk products and the variety of fresh fruits and vegetables in small and mid-sized WIC-authorized stores in Nashville/Davidson County, Tennessee, after the implementation of Final Rule changes in the WIC program in October 2014.

## Methods

We used a natural experimental design to evaluate the effect of the 2014 modification of the WIC program (the intervention) on the grocery store food environment. We analyzed secondary data to compare within-group changes from preintervention (March 2014) to postintervention (February 2016). The secondary data were initially collected for another purpose as part of the Nashville Children Eating Well (CHEW) for Health project, which was reviewed and approved by the institutional review board of Tennessee State University. The data used for our study were from grocery store audits that did not include human subjects; thus, informed consent was not required.

### Study setting and store sample

The WIC program of the local public health department provided the list of all WIC-authorized grocery stores in Nashville/Davidson County, Tennessee, of which 26 were designated by WIC as a small or mid-sized grocery store. The Tennessee WIC Vendor Handbook classifies stores into 3 size categories (small, medium, or large) based on the type of store (eg, small independent store, large independent store, or major chain) and the minimum number of vouchers the store is able to transact for many food categories (2 vouchers for small stores, 3 vouchers for medium stores, and 4 vouchers for large stores) (21). Store addresses were geocoded and mapped by using ArcGIS version 10.3 (Esri). All 26 small and mid-sized stores were invited to participate in the CHEW program, and 19 signed up to participate through written agreement, which included agreeing to allow the store to be visited by research personnel periodically to conduct brief audits of available foods. Seven stores declined. Subsequently, one participating store became ineligible for WIC, leaving 18 small or mid-sized WIC-authorized stores in the sample.

We collected data on the socioeconomic characteristics (education level, annual household income, race/ethnicity, access to a vehicle for transportation, and renter-occupied housing) of the census tracts in which the 18 stores were located and compared these data with data on 5-year county averages from the American Community Survey 2009–2013 (22). Most stores were located in census tracts that were above the county average on these measures of low socioeconomic status, and most were located in census tracts that had concentrations of low-income, African American, and Hispanic populations (Table 1). Stores that had Spanish-language names were classified as serving the Hispanic population; all others were classified as serving the general population. We used ArcGIS to create a map (Figure) showing the location of each participating store, a 3-mile–radial buffer zone around each participating store, and food deserts. Food deserts (ie, areas that lack full-service grocery stores) were identified by analyzing previously collected data on food desert scores (23). Our set of 18 stores provided excellent coverage for most areas considered to be food deserts.

**Table 1:** Socioeconomic Characteristics of the Census Tracts Where Participating WIC Stores Were Located, Nashville/Davidson County, Tennessee, 2009–2013^a^

Store	<High School Graduate	High School Graduate	Annual Household Income <$15,000	Annual Household Income <$20,000	Black	Hispanic	No Vehicle for Transportation	Renter-Occupied Housing
**Nashville/Davidson County, %**								
Mean	15.4	25.3	15.9	28.2	29.8	9.2	3.9	42.2
**Stores serving general population, %^b^**								
A	44.5	27.7	22.8	42.2	41.5	22.0	0.6	39.2
B	23.5	28.9	22.6	39.3	74.1	0.6	6.9	65.3
C	17.7	34.7	22.2	35.6	85.8	4.6	5.5	34.5
D	24.0	34.1	17.1	32.4	69.5	0.2	4.0	25.4
E	7.4	23.7	10.4	17.4	36.1	10.7	1.2	68.1
F	27.4	35.1	9.5	35.2	27.6	36.8	1.9	39.1
G	24.7	22.3	19.9	31.8	16.9	18.5	3.7	77.5
H	20.0	20.6	22.6	43.4	15.6	19.2	0.0	53.3
I	32.7	28.9	16.4	37.9	14.6	50.6	4.7	75.7
**Stores serving Hispanic population, %^b^**								
J	13.2	24.5	2.6	8.0	26.3	6.1	0.0	12.0
K	37.6	43.5	27.7	56.6	25.6	28.2	13.0	95.3
L	10.0	36.6	8.7	20.2	51.3	12.1	2.5	16.5
M	24.3	26.6	31.1	54.1	22.1	27.8	1.5	65.5
N	37.6	43.5	27.7	56.6	25.6	28.2	13.0	95.3
O	43.5	29.4	11.9	34.3	11.4	42.3	0.5	57.3
P	18.1	30.5	9.9	31.0	21.0	15.7	2.2	64.5
Q	26.4	37.1	5.2	20.4	14.0	32.4	1.3	44.9
R	11.5	29.2	9.7	24.3	28.0	21.0	0.5	25.3

Abbreviation: WIC, Special Supplemental Nutrition Program for Women, Infants, and Children.

a Data source: American Community Survey, 2009–2013 5-year averages (22).

b Stores that had Spanish-language names were classified as serving the Hispanic population; all others were classified as serving the general population.

**Figure:**
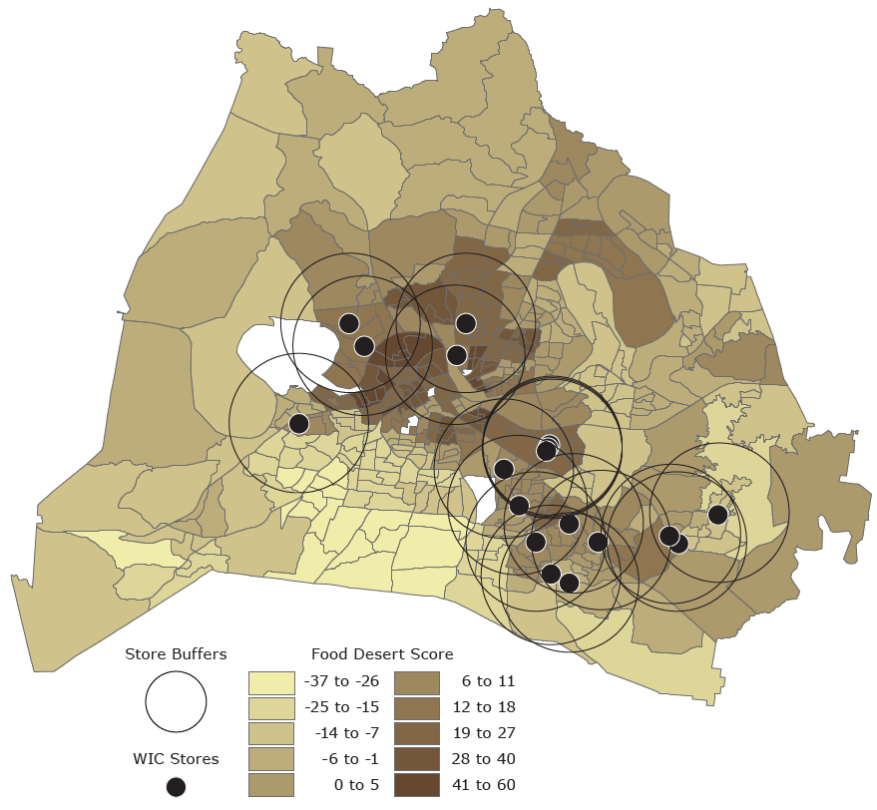
Location of 18 sampled WIC stores, by census-tract food desert score, Nashville/Davidson County, Tennessee. Each store is surrounded by a 3-mile-radial buffer. Food desert scores, ranging from −37 to 60, were grouped into 10 categories. The higher the score, the greater the likelihood of a food desert; a score of 20 or above indicates a food desert. The food desert score was created by summing 36 *z* scores of variables that measure distance to grocery store, distance to bus stops, social characteristics and poverty, race/ethnicity, chronic disease prevalence, and access to transportation (23). Food desert scores cannot be computed for census tracts that have no residential parcels; these tracts are shown in white.

### Measures

We conducted the grocery store audits using the validated Nutrition Environment Measures Survey in Stores (NEMS–S) tool (24) to evaluate the presence of a range of food products in the selected stores. We used the 5 sections of the 11-section NEMS–S that were relevant to the CHEW project: the sections on general store information, milk, fruit, vegetables, and bread. The NEM–S lists products in each section that are documented as available in various sizes (eg, gallon, half-gallon) and various kinds (eg, whole wheat bread, white bread). The NEMS–S allows for recording of prices and ratings of quality for fresh fruits and vegetables. 

**Milk shelf space.** The NEMS–S milk section prompts documentation of the amount of shelf space occupied by each type of milk product, measured as the number of slots in the refrigerator case for each type. Using this information, we calculated the proportion of total milk shelf space that was occupied by the following types of milk: nonfat (skim), low-fat (1%), 2%, whole, and other types (eg, soy milk). The primary outcome measure for milk was the proportion of nonfat and low-fat milk combined, but we also examined the proportion of nonfat milk and low-fat milk separately.

**Produce variety.** The NEMS–S fruit and vegetable sections list 10 common fruit items (bananas, apples, oranges, grapes, cantaloupe, peaches, strawberries, honeydew melon, watermelon, and pears) and 10 common vegetable items (carrots, tomatoes, sweet peppers, broccoli, lettuce, corn, celery, cucumbers, cabbage, and cauliflower). In addition, we asked observers to record all other fresh fruit and vegetable varieties that were stocked. We subsequently reviewed these lists to manually code each fruit or vegetable that was not already listed in NEMS–S, eliminated duplicates, and, where needed, incorporated items into NEMS–S categories. For example, red potatoes and Idaho potatoes were combined into a single category for potatoes; all types of onions were counted as a single category; collard greens, turnip greens, and kale were combined into one category for greens. The final list of manually coded “other” fruits included the following: blackberries, cranberries, dates, grapefruit, guava, kiwi, lemon, lime, mango, papaya, persimmon, pineapple, plum, pomegranate, raspberries, rambutan, and starfruit. Manually coded “other” vegetables included the following: asparagus, avocado, beans, beets, Brussels sprouts, cactus, cassava, chayote, edamame, eggplant, green beans, greens, jicama, malanga, mushrooms, olives, onions, peas, plantain, potatoes, radishes, rutabaga, spinach, sweet potatoes, turnips, yellow squash, and zucchini. Finally, the NEMS–S varieties and the additional manually coded varieties were added together. The primary outcome measure for produce was the total number of fruit and vegetable varieties available; we also examined the number of fruit items and vegetable varieties separately, as well as the number of “other” manually coded fruits and vegetable varieties.

### Data collection and analysis

Observers were trained in the use of the NEMS–S through the NEMS–S online training module and additional training using the NEMS–S manual. Two coders at a time were sent to each store and used a paper version of the NEMS–S to collect data. Coders also took digital photographs of the stores. Data were collected in March 2014 and February 2016.

Data were transferred from the paper forms to a database in SPSS version 24.0 (IBM Corporation), and the produce data were manually coded as described above. We calculated descriptive statistics, including means, standard deviations, and percentages. Paired-sample *t* tests were used to compare preintervention and postintervention means of the outcome variables to test hypotheses of differences in food availability before and after the 2014 Final Rule changes. Significance was set at an α of .05. Our hypotheses were that after implementation of the WIC Final Rule 1) the proportion of shelf space for low-fat and nonfat milk would increase, and 2) the variety of fresh fruits and vegetables would increase. We did not hypothesize a change in the availability of “other” types of milk (soy milk or tofu) products because only a small percentage of WIC participants, due to allergies or other medical reasons, are authorized to receive these items as milk substitutes, and thus these participants would likely not affect the demand for these products in stores after the October 2014 changes. 

## Results

In March 2014, 8 of 18 stores had only one or 2 cash registers; one store had one cash register, 7 stores had 2, 7 stores had 3, 2 stores had 4, and one store had 6. All participating stores accepted the Supplemental Nutrition Assistance Program (SNAP, formerly called the Food Stamp Program) in addition to WIC.

The mean percentage of shelf space occupied by low-fat and nonfat milk combined increased significantly, from 2.5% in 2014 to 14.4% in 2016 (*P* = .003) (Table 2). This increase was primarily driven by a significant increase in the proportion of shelf space for low-fat milk, which increased from 1.3% to 12.0% (*P* = .001). Meanwhile, the proportion of shelf space for nonfat milk was stable at 1.3% in 2014 and 2.4% in 2016 (*P* = .48). We found no significant differences in shelf space for milk between stores targeting Hispanic customers and stores serving the general population at either time.

**Table 2:** Milk Shelf Space and Fresh Produce Variety in 18 Sampled WIC Stores, Before (March 2014) and After (February 2016) WIC Final Rule Changes, Nashville, Tennessee

Variable	Before Changes, Mean (Standard Deviation)	After Changes, Mean (Standard Deviation)	*P* Value^a^
**Percentage of shelf space by milk type**			
Total nonfat or low-fat milk	2.5 (7.4)	14.4 (13.5)	.003
Total reduced-fat or whole milk	69.4 (23.5)	60.5 (23.3)	.20
Nonfat (skim)	1.3 (3.7)	2.4 (6.4)	.48
Low fat (1%)	1.3 (3.7)	12.0 (10.6)	.001
Reduced fat (2%)	34.4 (11.4)	33.3 (20.1)	.82
Whole	35.0 (13.1)	27.3 (12.8)	.06
Other (eg, nondairy alternatives)	28.1 (23.6)	25.1 (21.2)	.54
**No. of varieties of fresh produce**			
Fruit and vegetable varieties	24.3 (6.6)	27.7 (6.4)	.01
Fruit varieties	9.4 (3.0)	10.3 (2.9)	.14
Vegetable varieties	14.9 (4.2)	17.4 (4.3)	.008

Abbreviation: WIC, Special Supplemental Nutrition Program for Women, Infants, and Children.

a *P* values from paired-sample *t* tests.

The total number of fruit and vegetable varieties combined increased significantly from 24.3 in 2014 to 27.7 in 2016 (*P* = .01) (Table 2). When we examined fruit and vegetables separately, the number of vegetable varieties increased significantly from 14.9 in 2014 to 17.4 in 2016 (*P* = .008), but the number of fruit varieties did not (9.4 to 10.3; *P* = .14). Stores that targeted Hispanic customers had a greater variety of “other” fruits (5.9 vs 3.2;* P* = .006) and “other” vegetables (10.6 vs 8.0;* P* =.04) than did stores serving the general population in 2016, although we found no differences in March 2014.

## Discussion

The 2014 Final Rule included 2 major changes to WIC food packages: 1) allowing only nonfat and low-fat milk for mothers and for children aged 2 to 4 years (eliminating 2% milk), and 2) increasing the CVV for children from $6 to $8. Our retrospective natural experiment among small and mid-sized WIC stores in Nashville/Davidson County, Tennessee, suggests that these food-package changes may have produced 2 corresponding changes in the retail food environment in these stores. Namely, stores began stocking a larger relative share of low-fat (1%) milk in the refrigerator cases, and stores increased the variety of fresh vegetables offered in their produce departments. Given that the growing seasons for fruits and vegetables in Tennessee start in April or later in the spring and summer (25), we would not expect the variety of produce in the stores to have changed as a result of the difference in months of data collection (March vs February). In fact, the observed increases in variety are conservative estimates, because the data were collected for the second time in February, one month earlier than the data were collected the first time.

Our literature search in December 2016 showed that no other studies examined the effect of the 2014 Final Rule changes. However, numerous studies evaluated the effect of the 2009 Interim Rule changes, which included eliminating whole milk from the packages for mothers and children aged 2 or older and initiating the CVV for the first time, with $10 for mothers and $6 for children. A systematic review concluded that the rule changes in 2009 were associated with improvements in the quality of dietary intake among WIC participants, including greater consumption of reduced-fat milk and fruits and vegetables (26).

Six studies evaluated the effect of the 2009 changes on the retail food environment (8,10,11,13,27,28). Among these 6 studies, 2 were conducted in Connecticut, one in Philadelphia, one in Illinois around Chicago, one in Texas, and one in New Orleans, which was the only study in the southeastern region. Three of the 4 that examined the availability of reduced-fat milk observed an increase in shelf space for reduced-fat milk (8,11,13,27). Two studies assessed changes in the total number of fruit and vegetable varieties; one study found a significant increase (8), and the other found no change (28). Of the 3 studies that measured changes in the availability of fruits separately from changes in the availability of vegetables, 2 studies found a significant increase in fruit varieties only (13,27), while the other observed an increase in vegetable varieties only (11).

Our study findings were similar for the effect of the 2014 Final Rule changes, which eliminated 2% milk and increased the CVV value for children, on the retail food environment among small and mid-sized WIC-authorized stores in a mid-sized southern city. Similar to the 2009 changes, the 2014 changes led to greater availability of the targeted low-fat milk options and greater produce variety. Although previous studies reported mixed results on the variety of fruits and vegetables, our study showed that the increase in produce variety was driven by the availability of vegetables.

Our study had several limitations, including the small sample size of WIC-authorized stores in our study area. The sample size did not provide sufficient statistical power to conduct multivariate analyses to assess the possible moderating role of store characteristics in the effect of the 2014 program changes. It would have been preferable for both store audits to have taken place in the same month, although we do not believe that the one-month preseason difference affected our findings. Furthermore, the secondary data source we used did not include data on non-WIC grocery stores as a comparison group. Thus, we cannot conclude that the changes observed in the WIC stores from 2014 to 2016 were due to changes in the WIC food packages and not due to spurious factors. However, our study is the first to examine the effect of the 2014 Final Rule changes, and it was conducted in a southern city where little research on WIC program changes has been conducted. Thus, it contributes information to the literature on WIC program evaluation despite its limitations.

Our retrospective natural experiment among small and mid-sized WIC stores in Nashville/Davidson County, Tennessee, suggests that 2014 Final Rule changes in WIC food packages may have motivated stores to stock more low-fat (1%) milk and a greater variety of fresh vegetables than previously. Although the findings are not conclusive, they suggest that the 2014 changes may have had the intended impact of enhancing the effects of the changes that were started in 2009. Finally, our study highlights the need for further research to examine the effect of the 2014 Final Rule changes on the retail food environment in other cities as well as the effect on individual-level purchasing behavior and dietary intake. Future research could complement audits with qualitative data from store owners and managers to explore their reasons for making inventory changes, as well as conduct multiple visits to assess the sustainability of any changes in store inventory over time as the stores adjust to responses in consumer demand.
